# The KIF18A Inhibitor ATX020 Induces Mitotic Arrest and DNA Damage in Chromosomally Instable High-Grade Serous Ovarian Cancer Cells

**DOI:** 10.3390/cells14231863

**Published:** 2025-11-26

**Authors:** Jayakumar Nair, Tzu-Ting Huang, Maureen Lynes, Sanjoy Khan, Serena Silver, Jung-Min Lee

**Affiliations:** 1 Women’s Malignancies Branch, Center for Cancer Research, National Cancer Institute, National Institutes of Health, 9000 Rockville Pike, Bethesda, MD 20892, USA; tzu-ting.huang@nih.gov (T.-T.H.); leej6@mail.nih.gov (J.-M.L.); 2 Accent Therapeutics, Lexington, MA 02421, USA

**Keywords:** KIF18A, ATX020, HGSOC, ovarian cancer, metaphase, DNA damage

## Abstract

High-grade serous ovarian cancer (HGSOC) is the most common (~80%) and lethal ovarian cancer subtype in the United States, characterized by *TP53* mutations and DNA repair defects causing chromosomal instability (CIN). KIF18A is an essential cytoskeletal motor protein for cell division in CIN+ cancer cells, but it is not necessary for cell division in normal cells. Therefore, KIF18A represents a promising target for therapeutic interventions in CIN+ cancers. We investigated the use of a novel KIF18A inhibitor ATX020, for selectively targeting CIN+ HGSOC cells using growth inhibition assays, invasion assays, immunoassays, cell cycle analysis, and immunofluorescence techniques. Using DepMap and flow cytometry, we classified a panel of HGSOC cell lines based on aneuploidy scores (AS) and ploidy levels and identified a correlation between these classifications and sensitivity against ATX020. ATX020 induced cytotoxicity through mitotic arrest and DNA damage, and reduced tumor growth in HGSOC with high aneuploidy scores (AS). Mechanistically, ATX020 blocks KIF18A’s plus-end movement on spindle fibers, increasing spindle length, resulting in chromosomal mis-segregation, aneuploidy, and DNA damage. Our findings suggest that ATX020 inhibits CIN+ HGSOC cells mainly by inducing mitotic arrest and DNA damage, disrupting KIF18A’s function crucial for mitosis.

## 1. Introduction

High-grade serous ovarian cancer (HGSOC) is the most common subtype of ovarian cancer (~80%) and one of the most lethal gynecological malignancies in the United States [1]. HGSOC is characterized by universal TP53 mutations, genomic instability, and defects in DNA damage repair (DDR) pathways such as mutations in the BRCA1 and BRCA2 genes. Defects in DNA double-strand break (DSB) repair, particularly homologous recombination repair deficiency [2], make these cancers sensitive to DNA-damaging agents like platinum and PARP inhibitors (PARPis) [3,4]. The standard of care treatment for HGSOC includes optimal debulking surgery followed by platinum- and taxane-based combination chemo-therapy [5]. However, most patients are diagnosed at advanced stages, relapse and eventually develop resistance to conventional therapies, highlighting an unmet need for new druggable targets [6].

Multiple mechanisms mediate resistance to platinum- and taxane-based chemotherapies in gynecologic cancers. Among many, elevated levels of 10 cytoskeletal-associated proteins (KIF14, KIF20A, KIF18A, ASPM, CEP55, DLGAP5, MAP9, ANLN, SCIN, and CCDC88A) are linked to a malignant ovarian cancer phenotype and resistance to paclitaxel [7]. For example, KIF18A, a member of the kinesin motor protein family, drives an ATP-dependent plus-end accumulation on kinetochore MTs, essential for proper MT organization and chromosome congression during cytokinesis [8,9]. KIF18A is overexpressed in several cancers, and its high expression correlates with worse prognoses [10,11,12,13]. Schiewek et al. also reported that higher KIF18A expression in ovarian cancer tumors, compared to borderline tumors, is associated with late-stage presentation at diagnosis (FIGO IIIC-IV) and poor clinical outcome [7].

Chromosomal instability (CIN) is a continuous process describing the rate of loss or gain of whole chromosomes (ploidy) or chromosome segments (segmental aneuploidy) within the cell population due to errors during cell division [14]. These changes are often caused by defective microtubule dynamics and abnormal spindle assembly [15], which are frequently worsened by genetic alterations in TP53 and other cancer-related genes such as BRCA1, CCNE1, or RB1 [16,17]. The aneuploidy score (AS), a correlate of CIN, reflects the total number of chromosomal arms or segments lost or gained compared to the normal diploid state [14]. CIN is common in ~91% of individual and serial HGSOC samples taken from ascites, with higher levels of CIN observed in chemo-resistant disease [18]. Recently, Marquis and colleagues demonstrated that cancer cells with TP53 mutations and persistent CIN are susceptible to KIF18A inhibition, while normal, diploid, or near-diploid cells remain unaffected [19]. Additionally, the unstable microtubule dynamics in high-CIN cells make them more dependent on KIF18A’s ability to stabilize microtubule dynamics for survival, unlike normal cells [19]. This selective dependency makes KIF18A-targeted therapy a promising option for HGSOC, as cells associated with this cancer harbor genomic alterations linked to CIN [7,15].

Given the potential of KIF18A inhibition in ovarian cancer, we conducted preclinical evaluations of a novel KIF18A inhibitor, ATX020 to assess its activity in various HGSOC cell line models including platinum-resistant and PARPi-resistant cell line models [20]. We screened a panel of 11 HGSOC cell lines with different levels of ploidy and CIN to identify parameters that may correlate with sensitivity to ATX020. Additionally, we performed cellular and molecular studies to understand its effects on the mechanisms regulating microtubule dynamics and DNA repair.

## 2. Materials and Methods

### 2.1. Cell Lines

OVCAR3, OVCAR8, and SKOV3 cell lines were obtained from the NCI-60 collection at the National Cancer Institute (NCI), National Institutes of Health (NIH, Frederick, MD, USA). The cell lines A2780, TOV21G (#CRL-3577), and OV90 (#CRL-3585) were acquired from the American Type Culture Collection (ATCC, Manassas, VA, USA). JHOS4 (#RCB1678) was obtained from the Riken Cell Bank (Ibaraki, Japan), and OVSAHO was kindly provided by Dr. Benjamin Bitler from the University of Colorado, Colorado, USA. PEO1 (#10032308) and PEO4 (#10032309) were purchased from Sigma-Aldrich (St. Louis, MO, USA). All cell lines were cultured in RPMI1640 media with L-glutamine (#11875119, Life Technologies, Frederick, MD, USA), containing 10% FBS, 1000 U/mL penicillin/streptomycin, 1 mM sodium pyruvate, and 5 µg/mL of insulin derived from bovine pancreas (#I0516, Sigma-Aldrich). Platinum (cisplatin) and PARPi (olaparib) sensitivity profiles and BRCA1/2 mutation status for cell lines are shown in Table 1 [21,22,23,24,25,26,27,28].

### 2.2. Murine Models of HGSOC

Immunocompromised Balb/c nude 6–8-week-old female mice were obtained from GemPharmatech Co., Ltd. (Nanjing, China). OVCAR8 cells (1 × 10^7^ per mouse) were injected subcutaneously into the right flank and allowed to grow until an average volume of 150 mm^3^ was reached. The animals were then divided into cages of 3–5 mice each and administered treatment orally once daily for three weeks, with ATX020 (100 mg/kg) suspended in vehicle (10% DMSO, 90% corn oil) or with vehicle alone. Tumor volume (TV) and body weight measurements were taken biweekly for up to 3 weeks. The TV (mm^3^) is estimated using the formula: TV = (a × b^2^)/2, where “a” and “b” are long and short diameters of a tumor, respectively.

### 2.3. Cell Growth Assays

The XTT assay was conducted as previously described [29]. Plates were read on a BioTek SynergyHT™ plate reader (BioTek Instruments, Winooski, VT, USA) and analyzed using Gen5™ software V 3.04. Absorbance at 490 nm was recorded as absolute values (corrected for background) or relative to the untreated control. IC50 values were estimated using Graphpad Prism^®^ V 10 (GraphPad Software LLC, Boston, MA, USA).

### 2.4. Clonogenic Assays

Assays to evaluate colony formation in the presence or absence of ATX020 were conducted in 12-well plates in triplicate. After overnight incubation (5000 cells per well), cells were treated with ATX020 (0–1 µg/mL in normal media) at 37 °C. The media were replaced on day 3 with fresh media containing ATX020, and plates were assayed on day 7. Wells were washed with phosphate-buffered saline (PBS), fixed with 100% methanol for 10 min, and then stained with 0.5% crystal violet in 20% methanol for 20 min. Following washing in distilled water, the plates were imaged. Staining intensity was quantified using FIJI™ V 1.54d (also known as ImageJ™, NIH), and the average of triplicate wells was plotted as a line graph.

### 2.5. Flow Cytometry

DNA content measurement for ploidy estimation [30] and cell cycle analysis was performed as previously described [31]. In brief, cells were fixed in 70% ethanol for at least 1 h at 4 °C. They were then washed twice with PBS and resuspended in 200 µL of propidium iodide (PI) solution (25 µg/mL) along with 50 µL of RNase A solution (0.5 µg/mL). After incubating for 1 h at room temperature, acquisition was conducted using a BD FACScanto™ II (BD Biosciences, Franklin Lakes, NJ, USA), and the data were analyzed with FlowJo^®^ software V10.7.2 (FlowJo LLC, Ashland, OR, USA). For ploidy analysis, acquisition was consistently performed across all cell lines using the parameters established for the reference diploid cell line A2780 [32]. Ploidy was estimated by dividing the mean fluorescence intensity (FI) of the G1 peak of the target cell line by the FI of the G1 peak of the reference diploid cell line A2780. Flow cytometric analysis for viability was conducted using Annexin V-FITC and 7AAD after suspending cells in Annexin V binding buffer according to the manufacturer’s instructions (Biolegend, San Diego, CA, USA).

### 2.6. DepMap Analysis

AS and ploidy numbers for various cancer cell lines, including ovarian cancer cell lines, were primarily obtained from the OMICSglobalSignatures dataset (Release 25Q2), downloaded from DepMap (https://depmap.org (accessed on 24 July 2025)). Some AS and ploidy values not available from this source were also gathered from published literature and analyses conducted in this study (Table 1). The AS for the PEO1 and PEO4 cell lines was calculated as previously described [33], using in-house spectral karyotyping (SKY) analysis data [34]. Ploidy and AS for all cell lines were plotted using GraphPad Prism^®^ (GraphPad Software LLC).

### 2.7. Mitotic Arrest Assays

For flow cytometric analysis of the mitotic marker phospho-histone H3 ser10 (pHH3-S10), harvested cells were first permeabilized with 0.1% Triton X-100 in PBS, stained with a mouse α-pHH3-S10-Alexa Fluor^®^ 647 antibody (#558217, BD Biosciences, Franklin Lakes, NJ, USA), followed by fixation and PI staining, as described above for cell cycle analysis.

### 2.8. Matrigel Invasion Assays

Invasion assays were performed using 24-well BioCoat^TM^ Matrigel^®^ invasion chamber plates from Corning (ThermoFisher Scientific, Waltham, MA, USA) according to the manufacturer’s instructions. Briefly, 1 × 105 cells were plated in serum-free RPMI media, with or without ATX020, in the upper chamber. The lower chambers contained complete media with serum, also with or without ATX020. The plates were incubated for 48 h, after which the upper chamber was processed and stained with crystal violet following the manufacturer’s guidelines. The cells that invaded, stained violet, and were visible on the bottom surface of the insert membrane were counted and plotted.

### 2.9. Immunoblotting

Immunoblotting was performed as described [31]. Antibodies against KIF18A (#ab72417) and pATM-S1981 (#ab81292) were obtained from Abcam (Abcam Inc., Waltham, MA, USA). Other antibodies against pHH3-S10 (#9701), histone H3 (HH3) (#14269), pKAP1-S824 (#4127), KAP1 (#5868), γH2AX-S139 (#9718), pCDK1-Y15 (#4539), CDK1 (#9116) and GAPDH (#2118) were obtained from Cell Signaling (Cell Signaling Technology, Denvers, MA, USA). Blots were visualized and documented on an Odyssey™ Fc gel documentation system (LI-COR biosystems, Lincoln, NE, USA).

### 2.10. Immunofluorescence Microscopy

Cells were cultured in chamber slides (Ibidi USA Inc., Fitchburg, WI, USA) overnight before treatment with ATX020 for 48 h. The slides were stained for KIF18A as previously described [35]. In brief, the slides were fixed in 100% methanol (chilled to −20 °C) for at least 10 min, then rinsed in 50% methanol, followed by two washes in PBS. Next, the slides were blocked in a blocking buffer (composed of 2.5% FBS, 200 mM glycine, and 0.1% Triton X-100 in PBS) for 1 h. They were then incubated in a blocking buffer containing rabbit antibodies against KIF18A (#A301-080A, Bethyl Laboratories, Montgomery, TX, USA) for 1 h, protected from light. After a brief rinse in 0.1% Triton X-100 in PBS, the slides were washed twice in PBS. They were then incubated for an additional hour in the dark with blocking buffer containing mouse anti-α-tubulin-AF488 (#322588) and the secondary antibody goat anti-rabbit IgG-AF568 (#A-11036, ThermoFisher Scientific). The slides were washed twice with PBS before staining with DAPI (300 nM) for 4 min, also in the dark. Following three washes, the slides were mounted with ProLong Anti-Fade^®^ mountant (#P36980, ThermoFisher Scientific) and visualized using a Leica Stellaris 8 FLIM laser confocal microscope (Leica Microsystems, Deerfield, IL, USA). Images were analyzed using Fiji™.

### 2.11. Quantification and Statistical Analysis

Student’s t-test was used to assess statistical significance for paired groups of data, while an unpaired Mann–Whitney U test was utilized for murine studies (GraphPad Software LLC). Minimum sample sizes required for statistical significance were determined in consultation with biostatisticians. In all plots presented in this study, *p*-values less than 0.05 are considered significant and are indicated with asterisks: * *p* < 0.05, ** *p* < 0.01, and *** *p* < 0.001, unless stated otherwise in the figure legends.

## 3. Results

### 3.1. High Ploidy and as Are Associated with Sensitivity to KIF18A Inhibition in HGSOC

**Figure 1:**
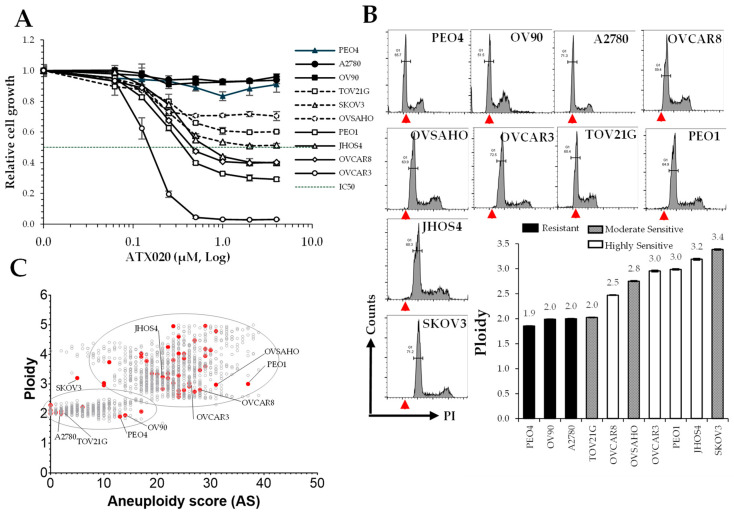
Sensitivity of HGSOC cells to ATX020 is partially influenced by ploidy and AS levels. (**A**) XTT assays assessed growth inhibition of 11 HGSOC cell lines after 72 h of ATX020 treatment at indicated concentrations. Growth inhibition was measured using XTT reagent. Mean absorbance (A490nm) values are plotted relative to untreated controls; error bars indicate standard deviation (SD) (*n* = 3). Zero drug concentration values were adjusted to a non-zero value to enable plotting on a logarithmic scale. Data is representative of 3 experimental repeats. (**B**) Flow cytometric analysis of the ploidy of HGSOC cell lines used in (**A**). Red arrowheads mark the diploid (2N) reference from A2780. Bar charts of ploidy (annotated), shaded by ATX020 sensitivity, are shown on the right. Data are representative of two independent experiments. (**C**) Scatter plot of AS and ploidy scores of all cancer cell lines were downloaded from the DepMap database. Ovarian cancer cell lines are marked in red, with study cell lines annotated.

A panel of 11 HGSOC cell lines with varying ploidy levels and AS was treated with increasing concentrations of ATX020 over 72 h, followed by XTT assays to assess growth inhibition. Based on the dose–response curves, cell lines were classified into three groups: highly sensitive, moderately sensitive, and resistant (Figure 1A and Table 1). IC50 values were calculated for the highly sensitive group, defined as those with >50% maximum growth inhibition compared to untreated cells. Silencing KIF18A with specific siRNAs recapitulated the inhibitory effects observed with ATX020 treatment (Supplementary Figure S1). To evaluate whether sensitivity correlated with CIN, we examined ploidy and AS in these cell lines. Ploidy was confirmed by flow cytometry using A2780 cell lines as a diploid (2N) reference control [33] (Figure 1B). Notably, higher ploidy levels were associated with increased sensitivity to ATX020 (Figure 1B).

When ploidy levels and AS were plotted for all cell lines in the dataset, including ovarian cancer cell lines, two distinct clusters emerged: a low ploidy/low AS cluster and a high ploidy/high AS cluster, indicating a positive correlation between ploidy and AS (Figure 1C), both of which are indicative of CIN [14,15,19]. Our results also suggest that ovarian cell lines with high sensitivity to ATX020 generally fell within the high ploidy/high AS cluster, whereas those with low sensitivity (resistance) (Table 1) tend to be in the low ploidy/low AS cluster (Figure 1C).

### 3.2. Growth Inhibition by ATX020 Was Primarily Caused by Its Effect on Tumorigenicity and Proliferation

**Figure 2:**
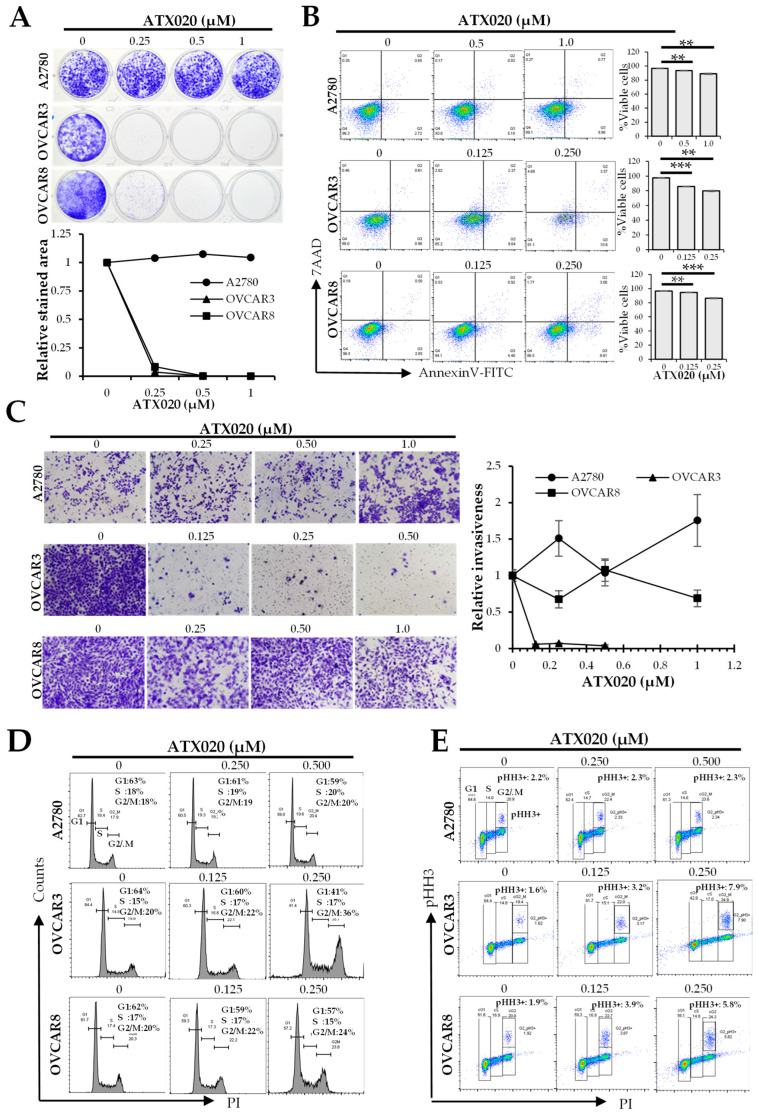
Effects of ATX020 on growth-related parameters in HGSOC cell lines. (**A**) Clonogenic assay on A2780, OVCAR3, and OVCAR8 treated with ATX020 for 7 days. Stained colony area was quantified using FIJI™, and plotted as mean ± SD (*n* = 3). (**B**) Flow cytometric analysis of cell viability after 72 h ATX020 treatment at sublethal concentrations. Data shown is representative of 3 experimental repeats. Bar charts display mean ± SD of % viable cells (*n* = 3). (**C**) Representative images of invasion assays (upper chamber membranes) in cells treated with or without ATX020 for 48 h. Line charts on the right show mean ± SD (*n* = 3) relative to controls. (**D**) Representative images of cell cycle analysis of ATX020-treated HGSOC cell lines stained with PI (*n* = 3). Histograms indicate G1, S, and G2/M populations using FlowJo. Data shown is representative of 3 experimental repeats. (**E**) Flow cytometric analysis of HGSOC cells treated with ATX020 over 48 h and stained for pHH3. Insert shows % pHH3+ cells relative to total cells. The different cell cycle phases are indicated as boxes. Data are representative of two experimental repeats. Significance was analyzed by Student’s *t*-test (** *p* < 0.01; *** *p* < 0.001).

To investigate how KIF18A influences HGSOC cell growth, we focused on three representative ovarian cancer cell lines (A2780, OVCAR3, and OVCAR8), given that they represent a range of ATX020 sensitivity and varying cisplatin and olaparib responses (Table 1). We next explored how various cell growth parameters, including clonogenicity, viability, and mitotic activity, affect sensitivity to ATX020. Clonogenic assays performed over 7 days of ATX020 treatment demonstrated marked growth inhibition in OVCAR3 and OVCAR8 cells, consistent with their high sensitivity in growth assays, whereas the resistant A2780 cells showed minimal growth inhibition (Figure 2A). Flow cytometry analysis of cell viability at IC50 concentrations over 72 h showed modest reduction in viable cells (~10–20%) across all three cell lines despite > 50% growth inhibition observed in XTT assays (Figure 2B). This finding suggests that KIF18A inhibition may exert a greater effect on tumorigenic potential and proliferative capacity than on short-term viability. Additionally, extending the incubation to 96 h did not change these results (data not shown).

We next examined whether ATX020-mediated growth inhibition was linked to impaired invasion capacity or cell cycle dysregulation. Invasion assays showed that OVCAR3, a platinum-resistant HGSOC cell line, was highly sensitive to ATX020, with >90% inhibition even at the lowest concentration tested of 0.125 µM. Conversely, both platinum-resistant OVCAR8 and platinum-sensitive A2780 displayed minimal inhibition of invasion capacity up to 1 µM of ATX020 (Figure 2C).

Consistent with prior reports that KIF18A inhibition induces mitotic arrest [17] ATX020 treatment led to a significant increase in the G2/M phase in OVCAR3 cells (36% vs. 20%), and a modest increase in OVCAR8 (24% vs. 20%) at the highest concentration tested (0.25 µM) of ATX020. In contrast, ATX020-resistant A2780 cells exhibited no significant change in cell cycle progression with ATX020 compared to untreated cells (Figure 2D). Most of the increased G2/M population was due to cells in mitotic arrest, as indicated by elevated pHH3+ G2/M fractions in OVCAR3 (7.9% vs. 1.6%) and OVCAR8 (5.8% vs. 1.9%) when gated on live cells (Figure 2E).

### 3.3. Effect of ATX020 on KIF18A Motor Function

**Figure 3:**
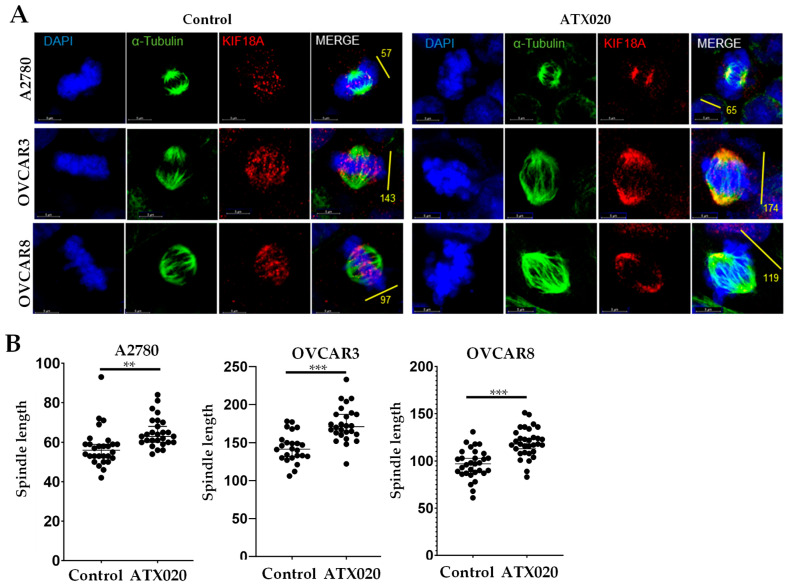
Impact of ATX020 on KIF18A motor function. (**A**) Immunofluorescence images of cells stained for KIF18A (red), α-tubulin (green), and nuclear stain DAPI (blue). Cells were untreated or treated with ATX020 (1 µM for A2780; 0.5 µM for OVCAR3 and OVCAR8) for 48 h before staining with the respective antibodies. (**B**) Spindle length (distance between spindle poles) was quantified in ≥ 25 metaphase cells per condition using FIJI™. Data are shown as mean ± SD (*n* = 3). Significance between treatments was analyzed by unpaired *t*-test (** *p* < 0.01; *** *p* < 0.001).

To investigate the effects of ATX020 on KIF18A mobility, we treated resistant A2780 and sensitive OVCAR3 and OVCAR8 cells with ATX020, then stained for microtubule component α-tubulin and KIF18A [35]. ATX020 caused accumulation of KIF18A closer to the spindle poles in both resistant and sensitive cells (Figure 3A) suggesting inhibition of plus-end movement of KIF18A. Additionally, measurement of the distance between spindle poles (spindle length) revealed that ATX020 significantly lengthened spindle fibers in cells regardless of their sensitivity to KIF18A inhibition (Figure 3B).

### 3.4. Crosstalk Between KIF18A Regulators and the DNA Damage Response

**Figure 4:**
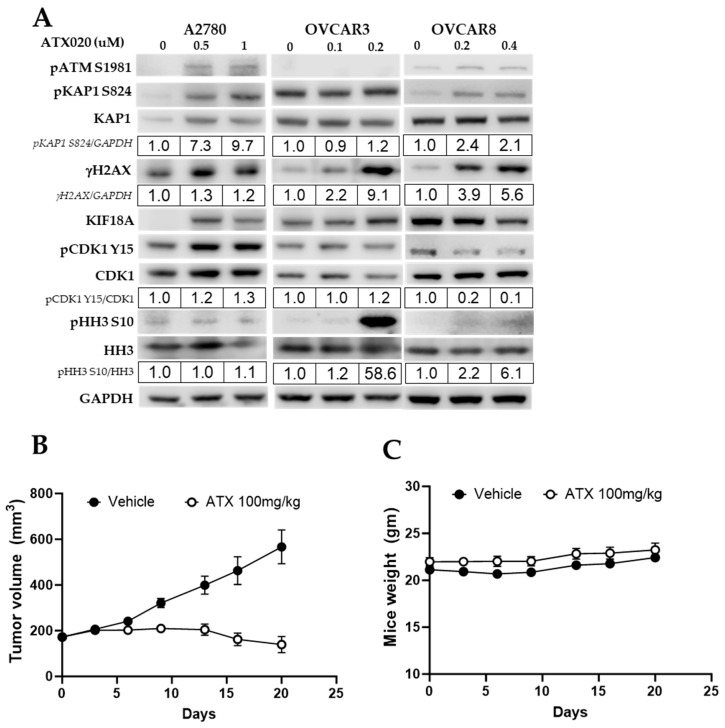
Activity of ATX020 in cell cycle regulation and in vivo tumor growth. (**A**) Western blot analysis of cell cycle and DNA damage markers from cell lysates with 24 h ATX020 treatment (*n* = 3). Phosphorylated proteins were quantified relative to total, normalized to untreated controls (set to 1), and values shown below blots. Values were rounded to two significant digits. Representative of 3 biologically independent experiments. (**B**,**C**) In vivo activity of ATX020 was tested in OVCAR8 subcutaneous xenografts. Tumor volumes (**B**) and mice weight (**C**) were measured weekly and plotted as mean ± SD (*n* = 5 per group). Significance was analyzed using the Mann–Whitney U test.

The activity of KIF18A on microtubule dynamics is regulated by the cell cycle regulator protein CDK1, which, in its active dephosphorylated form, phosphorylates and inhibits KIF18A when cells are not in metaphase [36]. WEE1 kinase inhibits CDK1 (pCDK1-Y15), while protein phosphatase 1 (PP1) activates it, controlling KIF18A activity to ensure proper chromosomal alignment metaphase [37]. In CIN+ cells, increased chromosomal stress from KIF18A inhibition often leads to greater DNA damage and higher levels of pHH3, a marker of mitotic arrest [19,36]. Hence, we examined upstream and downstream molecular events of DNA repair or cell cycle regulation to identify mechanisms underlying differential ATX020 sensitivity. Western blots showed higher expression of the DSB marker γH2AX-S139 in ATX020-sensitive OVCAR3 and OVCAR8 cells compared to untreated controls, while ATX020-resistant A2780 cells showed minimal change (Figure 4A). Of note, ATX020 (0.2 µM) induced roughly a 59-fold increase in pHH3 expression in OVCAR3 cells, whereas OVCAR8 showed a modest increase of 2- and 6-fold at 0.2 and 0.4 µM, respectively. A2780 cells did not exhibit notable changes in pHH3 levels even at 1 µM ATX020 (Figure 4A). These findings are consistent with the G2 and pHH3+ populations observed in cell cycle analysis following ATX020 treatment (Figure 2D,E).

### 3.5. In Vivo Activity of ATX020 in HGSOC Murine Models

The antitumor activity of ATX020 was evaluated in OVCAR8 xenograft models. Results showed that ATX020 significantly suppressed tumor growth, with statistical significance maintained (*p* < 0.05) even one week after treatment cessation (Figure 4B). No evidence of drug-related toxicity such as loss of body weight (Figure 4C), morbidity or pain was observed during the treatment period.

## 4. Discussion

KIF18A, a member of the kinesin-8 family of motor proteins, regulates cell division and the mitotic checkpoint [19,38]. Its motor domain helps it to move along spindle fibers, and is crucial for microtubule growth and proper chromosome congression during metaphase [39]. Disruptions of KIF18A activity can lead to abnormal chromosome segregation and aneuploidy, which is common in breast and ovarian cancers [5,10,15,35,40]. In CIN+ cancers such as HGSOC, characterized by high aneuploidy and CIN [7,17], KIF18A is critical for managing microtubule dynamics and ensuring accurate cell division [7,17]. It ensures accurate division and survival in ovarian cancer, handling CIN stress without affecting normal cells [17,41].

Although *TP53* mutations are often associated with CIN [17,35], we found no link between *TP53* status alone and sensitivity to ATX020 across 11 screened HGSOC cell lines, which is consistent with a previous report with another KIF18A inhibitor AM-1882 [35]. Indeed, we found that cells with low ploidy and low AS showed greater resistance to ATX020, indicating that these factors are dependable predictors of sensitivity to this compound. KIF18A expression varied among the cell lines, but no correlation was found with sensitivity. This matches findings from other groups as well [17]. Thus, our findings could have important clinical implications for designing therapeutic strategies with KIF18A inhibitors in a molecularly selected population.

Unlike normal cells, CIN+ cells have higher microtubule polymerization rates and altered spindle forces that can influence chromosomal segregation if not properly regulated [14]. In these cells, KIF18A maintains proper spindle tension by regulating microtubule polymerization, ensuring correct chromosome alignment and segregation [19,35,41,42]. Inhibiting KIF18A disrupts microtubule dynamics, reducing microtubule tension, which causes longer spindle fibers as shown by increased spindle length, leading to chromosomal mis-segregation and growth arrest [17,19]. Mechanistically, our study also showed that ATX020 acts similarly to other KIF18A inhibitors like AM-1882 and AM-5308 [19,35] by blocking KIF18A’s plus-end movement in both sensitive and resistant cells. Both resistant and sensitive cells show increased spindle length, suggestive of spindle tension, that should result in increased DNA damage [17,19]. However, this did not seem to affect the growth of resistant A2780 cells, suggesting that KIF18A activity is dispensable in cells with more stable microtubule dynamics. This is consistent with the idea that normal cells with stable microtubule dynamics can survive without KIF18A, while CIN+ cells depend on KIF18A for survival [19,35].

Of note, in immunoblots, resistant diploid A2780 cells showed increased KIF18A expression upon ATX020 treatment, possibly due to compensatory upregulation, but this observation requires further mechanistic investigation. Also, the resistant cell line A2780 did not display upregulation of γH2AX, a DNA damage marker, while it showed increased ATM and WEE1 activity as indicated by increased inhibitory phosphorylation of CDK1. We speculate that A2780, as a diploid cell line, responds to DNA damage by activating ATM, marked by increased phosphorylation of its specific substrate KAP1 (pKAP1-S824) [43,44], and by activating WEE1 [45]. Additional experiments in diploid cell lines are needed to understand the elevation of the ATM substrate KAP1 (pKAP1-S824) [43,46], and activation of WEE1, in the absence of elevated γH2AX. Interestingly, recent studies have shown that CIN can be induced when WEE1 activity is inhibited [37], independent of KIF18A indicating a potential role for WEE1 in sensitizing cells to KIF18A inhibition. Further mechanistic studies into the anti-tumor activity of ATX020 are needed in various HGSOC cell lines and mouse models. Possible directions include combining ATX020 with cell cycle regulatory agents such as WEE1 inhibitors that target the G2/M checkpoint and induce CIN.

## 5. Conclusions

Our studies show that in ovarian cancer cells, AS and ploidy levels are associated with sensitivity to ATX020. Mechanistically, ATX020′s effect on growth inhibition is mainly due to its negative impact on tumorigenicity and proliferation, with little contribution from its effect on viability, at least over the short term. However, over extended periods, prolonged proliferation arrest may eventually impact viability. Overall, our findings indicate that KIF18A inhibition has promising potential with multiple molecules currently in clinical trials, and variables like AS and ploidy can effectively predict CIN status for developing therapeutic strategies.

## Figures and Tables

**Table 1:** Sensitivity profile of ovarian cancer cell lines used in the study.

Sensitivity to ATX020	Cell Line	IC50 (µM)	% Viable @max C	Ploidy	AS ^$^	TP53 Status	CNV	BRCA Mutation	Olaparib Sensitivity	Cisplatin Sensitivity
Resistant	PEO4	ND	>80%	1.9	12 ^#^	LOF	-	BRCA2 GOF	R	R
	A2780	ND	>80%	2	2	Wt	L	Wt	S	S
	OV90	ND	>80%	2.0	13	LOF	L	Wt	S	S
Moderate	TOV21G	ND	>50%	2	2	Wt	L	Wt	S	S
	SKOV3	ND	>50%	3.4	5	Null	L	Wt	R	R
	OVSAHO	ND	>50%	2.7	31	LOF	H	BRCA2 LOF	S	S
High	PEO1	0.64	<50%	3.0	37 ^#^	LOF	-	BRCA2 LOF	S	S
	JHOS4	0.65	<50%	3.2	22 *	?	H	BRCA1 LOF	S	S
	OVCAR8	0.46	<50%	2.5	28	LOF	H	Wt	R	R
	OVCAR3	0.22	<90%	3.0	26	LOF	H	Wt	R	R

Wt, Wildtype; R, Resistant; “^$^”: AS scores obtained from Cohen-Sharir et al., Nature 2021. [33]; S, Sensitive; “-”: Not determined/unavailable; “#”: AS determined from SKYkaryotyping in the lab; MSI, Microsatellite Instability; S, stable; L, Low; H, High; “*”: Obtained from OMICS global signature release 25Q2, DEPMAP; “?”: Mutation of unknown significance; ND, Value not determined since viability at highest ATX conc was >50%.

## Data Availability

Data used in this study may be requested by writing to the corresponding author.

## Supplementary Materials

The following supporting information can be downloaded at: https://www.mdpi.com/article/10.3390/cells14231863/s1, Figure S1: Silencing KIF18A shows similar toxic effects as ATX020.

## Abbreviations

The following abbreviations are used in this manuscript:

HGSOC	High-grade serous ovarian cancer
CIN	Chromosomally instable
AS	Aneuploidy score
KIF18A	Kinesin Family Member 18A
DDR	DNA damage repair
PARP	Poly(ADP-Ribose) Polymerase
FIGO	International Federation of Gynecology and Obstetrics
NCI	National Cancer Institute
NIH	National Institutes of Health
PBS	Phosphate-buffered saline
PI	Propidium iodide
FI	Fluorescence intensity
DSB	Double-strand break
DNA	Deoxyribo nucleic acid
XTT	2,3-Bis-(2-Methoxy-4-nitro-5-sulfophenyl)-2H-tetrazolium-5-carboxanilide
RNase	Ribonuclease
FBS	Fetal bovine serum
DAPI	4′,6-diamidino-2-phenylindole
GOF	Gain of function
LOF	Loss of function
