# Supplementary material for: The KIF18A Inhibitor ATX020 Induces Mitotic Arrest and DNA Damage in Chromosomally Instable High-Grade Serous Ovarian Cancer Cells
Source: Cells. 2025 Nov 26;14(23):1863. doi: 10.3390/cells14231863 (PMC12691420; doi:10.3390/cells14231863)
Supplement: Supplementary file 1 [file cells-14-01863-s001.zip › cells-3926247-supplementary.pdf]

Supplementary Methods

Small interfering RNA (siRNA) transfection. A pool of 4 specific siRNAs (OnTargetPlus™ smartpool) against KIF18A (#L-006849-00-0005, Horizon discovery, CO, USA) or scrambled siRNA (control siRNA) was used for transfection with Dharmafect-1 transfection reagent (#T-2001-01, Thermo Fisher Scientific) as per manufacturer's protocol. Cells were transfected for at least 48 hours before use for growth assays.

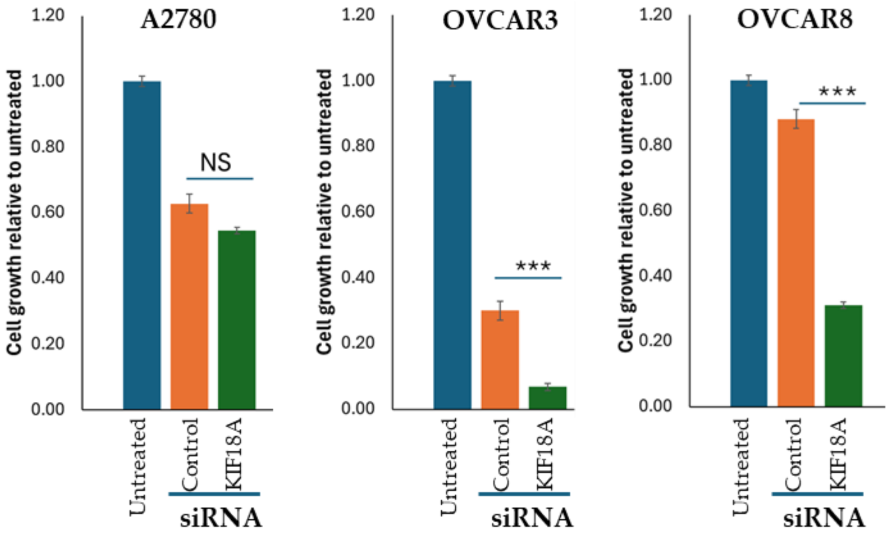

Supplementary Figure S1. Silencing KIF18A shows similar toxic effects as ATX020. HGSOC cells were transfected with either scrambled siRNA control or KIF18A-specific siRNAs for 48 hours, then seeded in 96-well plates and cultured for 72 hours before XTT assay. Bar charts show mean ± SD (n=3). Data shown are representative of 3 experimental replicates. Significance was analyzed using the Student's *t*-test (***$p < 0.001$).
